# Changing Patterns of Human Anthrax in Azerbaijan during the Post-Soviet and Preemptive Livestock Vaccination Eras

**DOI:** 10.1371/journal.pntd.0002985

**Published:** 2014-07-17

**Authors:** Ian Kracalik, Rakif Abdullayev, Kliment Asadov, Rita Ismayilova, Mehriban Baghirova, Narmin Ustun, Mazahir Shikhiyev, Aydin Talibzade, Jason K. Blackburn

**Affiliations:** 1 Spatial Epidemiology & Ecology Research Laboratory, Department of Geography, University of Florida, Gainesville, Florida, United States of America; 2 Emerging Pathogens Institute, University of Florida, Gainesville, Florida, United States of America; 3 Republican Anti-plague Station, Baku, Azerbaijan; 4 State Veterinary Service, Baku, Azerbaijan; Swiss Tropical and Public Health Institute, Switzerland

## Abstract

We assessed spatial and temporal changes in the occurrence of human anthrax in Azerbaijan during 1984 through 2010. Data on livestock outbreaks, vaccination efforts, and human anthrax incidence during Soviet governance, post-Soviet governance, preemptive livestock vaccination were analyzed. To evaluate changes in the spatio-temporal distribution of anthrax, we used a combination of spatial analysis, cluster detection, and weighted least squares segmented regression. Results indicated an annual percent change in incidence of +11.95% from 1984 to 1995 followed by declining rate of −35.24% after the initiation of livestock vaccination in 1996. Our findings also revealed geographic variation in the spatial distribution of reporting; cases were primarily concentrated in the west early in the study period and shifted eastward as time progressed. Over twenty years after the dissolution of the Soviet Union, the distribution of human anthrax in Azerbaijan has undergone marked changes. Despite decreases in the incidence of human anthrax, continued control measures in livestock are needed to mitigate its occurrence. The shifting patterns of human anthrax highlight the need for an integrated “One Health” approach that takes into account the changing geographic distribution of the disease.

## Author Summary

Zoonotic diseases, such as anthrax, represent a threat to public and veterinary health in many developing parts of the world. Control of anthrax is dependent upon several factors, including proper management of outbreaks and livestock vaccination. Following the dissolution of the Soviet Union, resources for disease management in Azerbaijan were dramatically diminished leading to increases in zoonotic diseases. In this study, our objective was to analyze human anthrax incidence during Soviet governance, post-Soviet governance, and after the implementation of a preemptive livestock vaccination campaign to identify potential changes in the occurrence of the disease. We applied spatial and temporal statistical approaches in a geographic information system to describe changes. Our findings provide evidence of a changing incidence and a shift in the geographic patterns of human anthrax. These findings highlight the importance of proper livestock disease management to mitigate human disease and the need for dynamic surveillance that takes into account changes in the distribution of disease.

## Introduction

The collapse of the Soviet Union in 1991 brought about profound alterations to disease management [1], [2], [3]. Once part of the largest public health system in the world, newly independent states (NIS) were suddenly faced with steep cuts in funding for disease surveillance and control [4]. The impacts of the deteriorating public health infrastructure were evident almost immediately as vaccine preventable diseases such as diphtheria increased dramatically [5], [6]. Similarly, as resources for veterinary surveillance dwindled, and new policies shifted agricultural production from state collectivization to individual ownership, surveillance efforts for animal health was severely diminished. Subsequently, there were reports of an increased incidence of zoonotic diseases (transferable from animals to humans), such as anthrax, in the former Soviet Union (FSU) [7], [8], [9]. Although more than twenty years have passed since Soviet independence, the status of anthrax in humans and livestock remains a concern across much of the FSU [8], [10], [11].

Anthrax is a soil-borne, bacterial zoonosis that is considered a threat in areas with a weakened public health system [12], [13], [14]. The disease primarily occurs in herbivores, with humans often secondarily afflicted through contact with infected animals or contaminated materials during agricultural activities such as slaughtering livestock [15], [16]. In rural areas and in populations dependent upon livestock for sustenance, anthrax can result in substantial economic losses from livestock mortality and lost worker productivity [17]. Effective control of the disease in humans is highly dependent upon mitigating the disease in animals through vaccination campaigns and proper outbreak management [17]. While the disease has been well managed in developed countries, anthrax persists in areas of sub-Saharan Africa, Southeast Asia, and parts of the former Soviet Union. The benefits of a “One Health” approach aimed at integrating animal and human health have been well established for zoonoses such as rabies and brucellosis [18], [19], [20], [21]. In Mongolia, the adequate provision of brucellosis livestock vaccination was shown to have mutual benefits to both the agriculture industry and human health [19]. Despite, the apparent benefits of a “One Health” approach for anthrax there is limited research examining the benefits of anthrax livestock control in reducing human incidence.

Anthrax is considered sporadic in Azerbaijan, although it is bordered by endemic and hyperendemic countries. The disease has a long history in the region with the first description of an anthrax like illness in the Caucasus recorded in 1697 [22]. In 1996, Azerbaijan experienced a series of livestock anthrax outbreaks that resulted in over 400 animal cases and more than 70 human cases [23], leading to the establishment of a more comprehensive preemptive livestock vaccination program. Recently, anthrax has reemerged in the Caucasus (Georgia) raising concerns over the potential trans-boundary spread of the disease [14], [24].

Using spatial and temporal analyses may allow for a better understanding of anthrax epidemiology and aid in disease management. A recent study in the country of Georgia used spatial statistics to identify areas of anthrax persistence in order to identify surveillance priorities [14]. In Azerbaijan researches used spatial-temporal statistics to analyze the distribution of human brucellosis [25]. They found evidence of a shifting geographic pattern in reporting that necessitated dynamic surveillance strategies. The purpose of this current study was to analyze and describe the historical distribution of human anthrax in Azerbaijan to identify possible spatial and temporal changes in the incidence of the disease. This study aimed to provide a better understanding of the occurrence of anthrax, identify areas to prioritize control, and aid in disease management.

## Methods

### Study Area

Azerbaijan is a geographically diverse country located in the south Caucasus with a population in the year 2012 of ∼9,295,800 [26]. The country is bordered by the Caspian Sea to the east, Iran to the south, Armenia and Turkey to the west, as well as Georgia and Russia to the north. Agriculture comprises an important aspect of the economy with approximately 38% of the population employed in agriculture and a majority (>90%) of livestock held by households or private owners [26]. There were an estimated 1,180 combined head of cattle, sheep, and goats per 1,000 persons [26].

### Descriptive Analysis

Under the governance of the FSU, anthrax was a nationally reportable infectious disease. After independence in December of 1991, Azerbaijan continued compulsory reporting of the disease with human surveillance and diagnostics carried out by the Anti-Plague Station (APS), which responds to health inquiries in order to obtain laboratory samples and confirm any diagnosis.

Data from 1984–2010 on all records of confirmed human cutaneous anthrax (referred to as human anthrax from here on) included the district of residence (presumed location of infection). The major time periods of interest corresponded to the periods of Soviet governance (1984–1991), post-Soviet governance before a nationally sponsored preemptive livestock vaccination program (1992–1995), and after the preemptive livestock vaccination campaign (1996–2010). The data were grouped into 4-year periods to visualize spatial changes and minimize the effects of population changes over time that may affect cumulative incidence rate estimations. Cumulative incidences per 100,000 persons (total cases/median population) were calculated for each of the seven 4-year periods and mapped at the district level. The last time period 2008–2010 only contained three years of data. Population estimates were obtained from the Republican APS in Baku and the Azeri State Statistical Committee (http://www.azstat.org/). In order to maintain a standard comparison between time periods, choropleth maps were binned into the following categories per 100,000 persons: 0, 0.1 to 2.0, 2.1 to 6.0, 6.1 to 14, and >14. A boxplot was used to visualize the distribution of human anthrax incidence among time periods. The number of livestock outbreaks (an outbreak was defined as 1 or more livestock cases) per year were also recorded and plotted across time with the total number of preemptive livestock anthrax vaccine doses administered by year. Recording of preemptive livestock vaccine did not begin until the year 1996. All maps were produced in ArcGIS 9.3.1 (ESRI, California) and statistical analyses were carried out in IBM SPSS 21 (IBM, New York).

### Mean Geographic Center

Movement in the geographic center of disease incidence, used as an exploratory spatial analysis tool, can provide insight into how the spatial distribution of reporting changed over time. We examined the yearly mean geographic center of human anthrax incidence at the district level for each of the 27 years in the study period using ArcGIS 9.3.1. The mean geographic center was derived using the following formula:
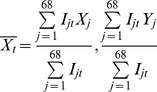
where the geographic center of incidence in the *j*th district is represented by a longitudinal and latitudinal coordinate *X*_j_, *Y*_j_. For each year *t*, let the anthrax incidence rate (per 100,000) in the *j*th district be denoted *I*_jt_. The mean geographic center of anthrax incidence in the *j* = 1,2,‥,68 districts at time *t* is then located at 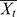 and 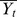. The latitude and longitude of the mean geographic center were then mapped for each time period and displayed with boxplots.

### Spatial Clustering

We used the spatial scan Poisson model in SaTScan v9.0 to identify districts with an increased incidence of anthrax [27], [28]. SaTScan has been described in detail elsewhere [27], [28], [29]. Briefly, the statistic uses a series of moving windows of varying size to identify the most likely clusters on the landscape by calculating the ratio of observed to expected observations and the likelihood function for each cluster. A measure of relative risk is then calculated by comparing the risk inside of the clusters to outside of the clusters. Models were run for each 4-year period using the centroid of each district and the total number of cases as well as the median year population of each time period. A maximum spatial limit of up to 30% of the population at risk was used allowing for no geographic overlap of the clusters and selecting clusters with p-values≤0.05.

### Piecewise Regression

Temporal trends in the annual incidence of human cutaneous anthrax (1984–2010) were evaluated using a piecewise regression approach implemented in the Jointpoint Regression Program v4.0.1 (http://surveillance.cancer.gov/joinpoint/) [30]. The piecewise regression model allows for the detection of temporal trend variations, such as increasing and decreasing trends in the occurrence of a disease over a period of time. Given the dramatic changes that followed the dissolution of the Soviet Union, we were interested in identifying any potential changes in the annual incidence of human anthrax. We used a log transformed dependent variable (log(x+1)) in a linear segmented regression to improve the fit of the model and allow for the estimation of yearly changes in percentages, which takes the form: log(R_t_+1) = μ(t)+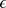
(t) where log(R_t_+1) is the natural log of the incidence rate in year *t* plus one and 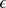 (t) is the residual for year *t* [30], [31]. To adjust for heteroscedastic errors (non-constant variance), we used a weighted least squares (WLS) approach with weights applied to each observation following Kim et al. [30]: *w* = *y* ^2^/*v* where *y* is the dependent variable and *v* is the square of the standard error of the dependent variable at each observation [30]. The piecewise regression mean μ(t) is described by a combination of (k+1) linear segments over a defined period of time and is written following Kim et al. [30]:
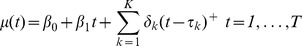
where the coefficient *δ_κ_* represents the change in slopes of the regression line segments. Changing rates in the data are indicated by breakpoints that signify changes in the slope of the regression line and are calculated using a permutation test to identify the most parsimonious model based on a range of possible breakpoints. The model was specified to allow for 0–5 possible breakpoints across the study period 1984–2010 (*T* = 27). For each possible regression line segment provided by the best fit model, the rate of change per year is given by the annual percent change (APC), which takes the form: *APC_i_* = { (*Exp*(*b_i_*)-1)}, where *b_i_* is the slope of the line segment [30], [31].

## Results

**Figure 1:**
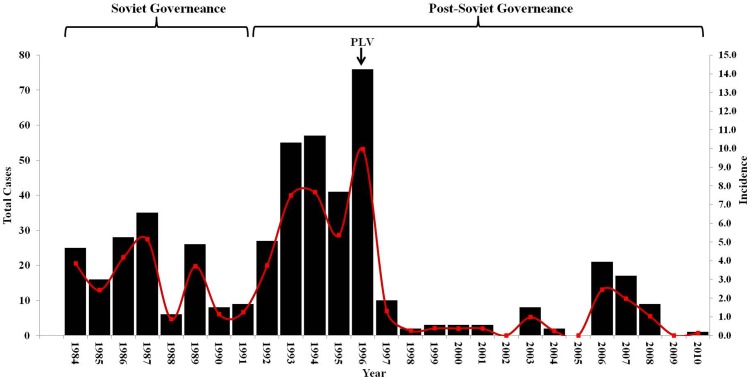
Annual human cutaneous anthrax cases in Azerbaijan during 1984 to 2010. Black bars represent total cases and the red line represents the incidence per 1 million population. The period of Soviet governance corresponds to the years 1984 to 1991, the period of post-Soviet governance before preemptive livestock vaccination corresponds to the years 1992 to 1995, and the initiation of preemptive livestock vaccination (PLV) corresponds to the years 1996 to 2010.

**Figure 2:**
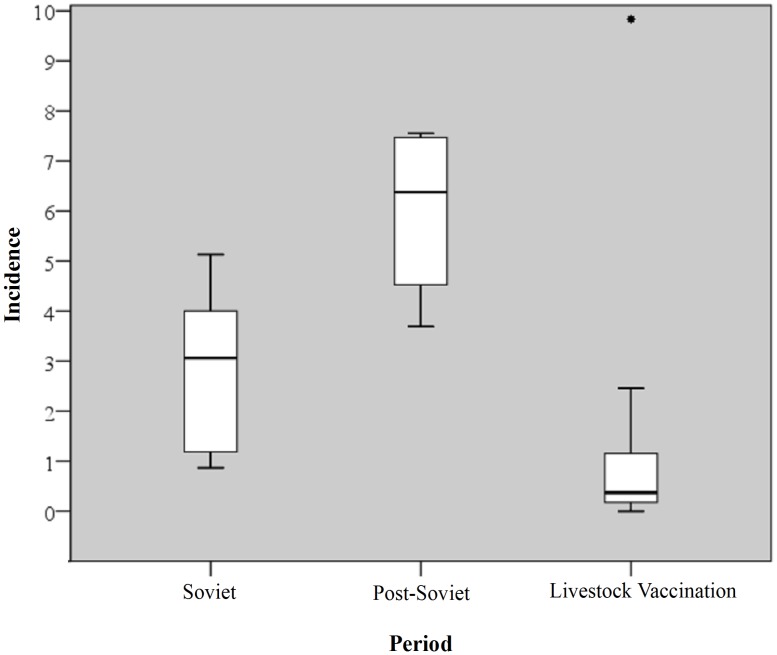
Boxplots of human cutaneous anthrax incidence at the district level grouped by the Soviet period (1984–1991), the post-Soviet period (1992–1995), and the period of preemptive livestock vaccination (1996–2010).

**Figure 3:**
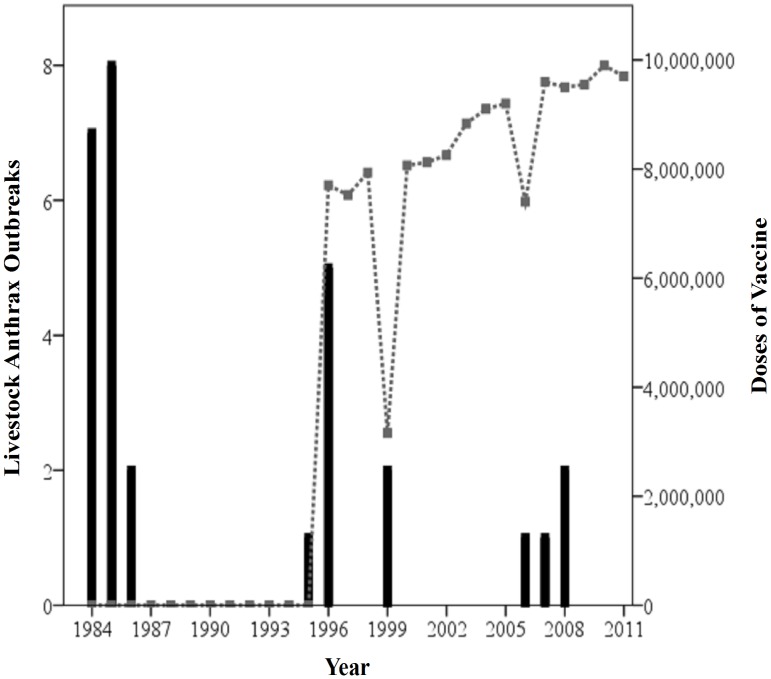
Graph displays the number of yearly livestock outbreaks of anthrax (black bars) and the total number of preemptive doses of livestock vaccine administered (grey line) during the period 1984–2010.

Figure 1 shows the national totals of human cutaneous anthrax per year during the Soviet period, post-Soviet, and livestock vaccination periods. There were a total of 489 [range: 0–76] reported cases of human cutaneous anthrax yielding a yearly mean of 17.46 (95% CI: 9.75, 25.16) cases. The annual incidence per 100,000 persons ranged from a low of 0 (95%CI: 0, 0.04) in the years 2002, 2005, and 2010 to a high of 1.01 (95% CI: 0.79, 1.23) in 1996. A boxplot shows the distribution of incidence between periods (Figure 2). The highest median incidence of human cutaneous anthrax per 100,000 persons occurred during the post-Soviet era 6.38 (95% CI: 4.53, 7.47) compared to 3.06 (95% CI: 1.25, 3.93) during Soviet-governance and 0.37 (95% CI: 0.25, 0.97) during preemptive livestock vaccination period. Figure 3 displays the number of livestock outbreaks over time with the total number of anthrax vaccine doses administered to livestock.

**Figure 4:**
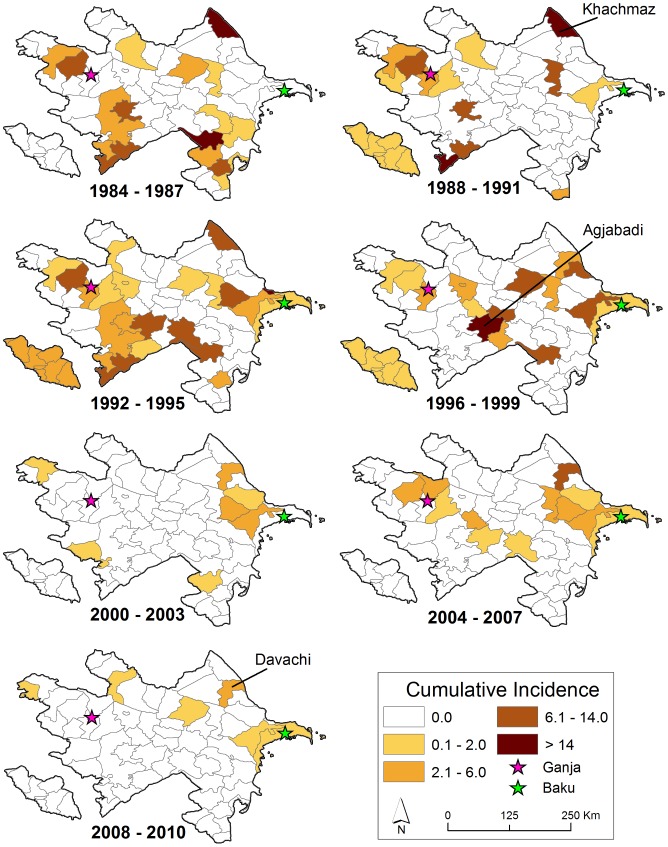
Cumulative Incidence of human cutaneous anthrax per 100,000 persons at the district level grouped into seven 4-year periods. District names correspond to peak cumulative incidences during the Soviet (1984–1991), post-Soviet (1992–1995), and preemptive livestock vaccination (1996–2010) periods.

**Figure 5:**
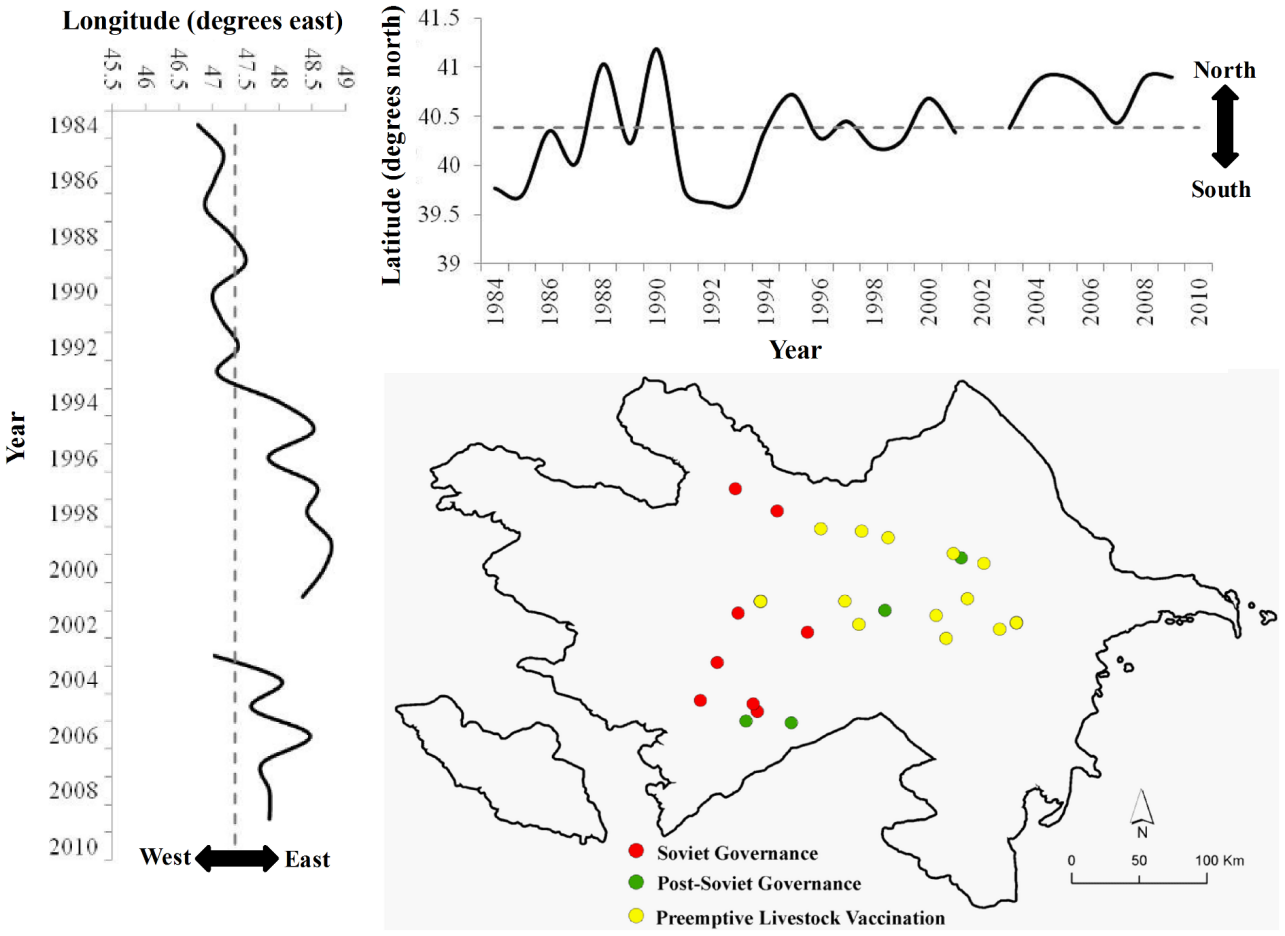
Weighted mean center of the yearly district incidence of human cutaneous anthrax. Graphs show the concentration of reporting by latitude and longitude over time. Map inset displays the location of the mean center by time period. Red dots represent the period of Soviet governance, green dots represent the period of post-Soviet governance, and yellow dots represent the period of preemptive livestock vaccination.

**Figure 6:**
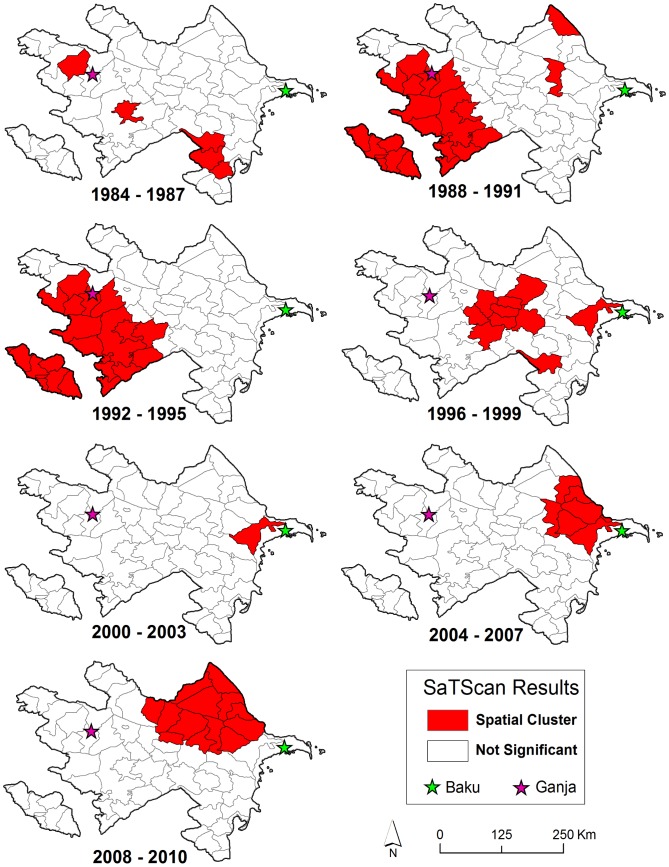
Maps show the results of the SatScan spatial scan statistic. Districts in red represent significant spatial clusters.

Cumulative incidence per 100,000 persons at the district level is displayed by time period in Figure 4. The highest reported cumulative incidence per 100,000 persons of 41.67 (95% CI: 16.21, 107.1) was in Khachmaz during 1987 to 1990 (Soviet Period). During the post-Soviet period the highest reported cumulative incidence per 100,000 persons was 18.91 (95% CI: 11.96, 29.88) in Agjabadi [1995 to 1998] compared to a high of 8.23 (95% CI: 3.2, 21.16) in Davachi [2003 to 2006] during the livestock vaccination period. Examination of the mean center of yearly incidence indicated a spatial shift in anthrax reporting over time (Figure 5). The incidence of human cutaneous anthrax was more concentrated in the west and south early in the study period during Soviet Governance. In the mid-1990s the burden of disease apparently shifted eastward towards the capital Baku. Incidence then remained primarily concentrated in eastern and northern Azerbaijan throughout the preemptive livestock vaccination period (Figure 4). Boxplots illustrate the differences in the latitudinal and longitudinal position of the mean center between time periods (Figure S1). The distribution of spatial clusters defined with the spatial scan statistic further illustrated the shifting geographic concentration of the disease (Figure 6). Clustering of human anthrax was identified in each of the seven 4-year periods. A greater number of clusters were detected in the west and south early in the study period, followed by a shift in the number of clusters eastward as time progressed.

**Figure 7:**
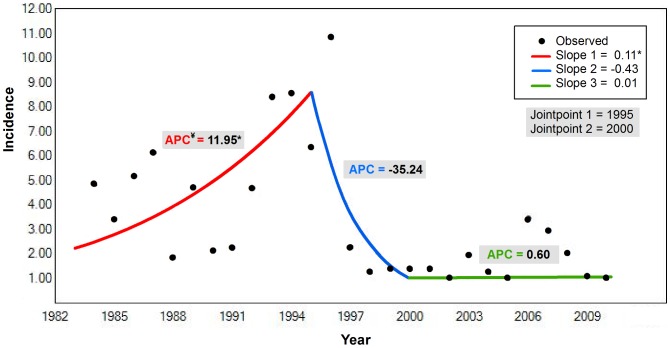
Results of the jointpoint regression analysis. Graph displays the 3-piece model with breakpoints at the years 1995 (95% CI: 1994, 1999) and 2000 (95% CI: 1999, 2006). The y-axis is the incidence per 1 million plus one and the x-axis is the year. The * indicates a significant slope of the line segment at the p = 0.02 level of significance. ^¥^Signifies a significant annual percent change in the rate of incidence for the line segment shown in red.

The piecewise regression indicated a 3-piece model was appropriate with breakpoints at year 1995 (95% CI: 1994, 1999) and 2000 (95% CI: 1999, 2006) (Figure 7). The slope coefficient was 0.11 (*p* = 0.02) for the period 1984 to 1995, −0.43 (*p* = 0.07) for the period 1995 to 2000, and 0.01 (*p* = 0.22) for period 2000 to 2010, which corresponded to an APC in rates of 11.95%, −35.24%, and 0.60%, respectively.

## Discussion

Results from our analyses of human anthrax in Azerbaijan indicated spatial and temporal changes during the last 27 years. This is consistent with previous research that has documented alterations in the occurrence of zoonotic diseases following the dissolution of the Soviet Union [7], [8], [25], [32], [33]. Our study revealed two broad patterns: an increasing trend in anthrax during Soviet governance up through the mid-1990s, followed by a decline in the number of reported cases subsequent to livestock vaccination and a geographic shift in the distribution of the disease eastward. These findings highlight the importance of dynamic disease management in livestock using a “One Health” approach to mitigate human disease and have important implications for future surveillance and the implementation of public health interventions [19].

The increasing trend in anthrax incidence of 11.95% annually identified during 1984 to1995 (Figure 7) is consistent with a breakdown in health measures and the establishment of new agricultural policies after the Soviet collapse [34]. Veterinary health control was the obligation of the state under Soviet governance, however, after independence the burden shifted to the private citizens who were often unwilling or unable to pay for services [35]. This, coupled with agricultural decollectivization, which shifted livestock ownership to the individual, rendered adequate disease management untenable. Similar increases in zoonotic diseases have been documented in Kazakhstan, Kyrgyzstan and other NIS [8], [32], [33], [36]. Conversely, our segmented regression model indicated a decreasing trend in incidence of −35.24% around the initiation of preemptive livestock vaccination. This finding is in line with studies that have documented a reduction in the occurrence of human anthrax following livestock vaccination campaigns in Cyprus and elsewhere [37], [38].

A dramatic increase in human anthrax in the Caucasus (Georgia) has highlighted the difficulty in controlling anthrax [24] due to factors such as noncompliance to livestock vaccination, inadequate vaccine coverage, and poor outbreak management. Control strategies for anthrax often rely on reactive vaccination to outbreaks after livestock or wildlife are found dead [39], [40]. While this form of control is effective for controlling an ongoing outbreak [40], routine vaccination in endemic areas is recommended [41]. Over 9 million doses of vaccine were administered in Azerbaijan during 2010. Generally, livestock vaccine is administered annually, but in some cases may include a booster in endemic areas where the risk is considered high [17]. During the study period livestock reporting was out of sync with human cases indicating a reliance on humans as sentinels for anthrax surveillance. The culture of slaughtering sick and dying animals to mitigate economic losses often skews livestock reporting, making targeted control difficult, while also exacerbating the risk of human infection [42], [43], [44]. Similar discrepancies between livestock and human reporting were also documented elsewhere in the region [14], which can be costly and ultimately impede control measures [8].

Several reasons are likely for the clustering of human anthrax identified in this study. First, heterogeneity in the occurrence of human anthrax is consistent with observations in livestock and wildlife, which are often the source of human infection [11], [45]. Second are the environmental constraints on the bacterium that limit its geographic distribution [17]. Previous models of *B. anthracis* have shown that the extent of its distribution is constrained by a combination of environmental factors [46], [47]. Similarly, in Georgia persistence of human cutaneous anthrax was shown to be related to soil pH as well as other environmental and anthropogenic factors [14]. Thus, disease transmission and persistence are more likely to occur in landscapes that promote pathogen survival. Third is the socio-cultural landscape. Occupation and cultural practices that increase peri-domestic contact with livestock such as animal husbandry and subsistence agriculture, which includes the practice of slaughtering sick animals can increase the risk of transmission [48]. In this study, clustering occurred during each decade and our findings suggested an eastward movement in reporting. One plausible explanation is that geographic changes in agriculture have influenced the distribution of anthrax reporting. Alterations to agriculture production have been implicated in the emergence of other zoonotic diseases such as Nipah virus [49], [50]. In Azerbaijan, the transition to independence resulted in the spatial restructuring of agricultural production in the east brought on by the abandonment of government controlled collective farms [51]. This finding is consistent with previous research that suggested a restructuring of agricultural production was a possible driver of a shift in the distribution of brucellosis from western to eastern Azerbaijan [25].

SaTScan has been widely used to investigate the space-time occurrence of infectious diseases [27], [28], [29]. We used SaTScan models with a maximum spatial extent of 30% of the population at risk, however there is little guidance on how to choose an appropriate threshold. There was little to no difference in SaTScan results using varying thresholds of 50%, 40%, and 30% of the population at risk (results not shown). In this study, we were particularly interested in identifying potential changes in the distribution of reporting over time; therefore we combined the spatial analytical approaches with a segmented regression. Although the weighted mean center analysis does not test any particular hypothesis or provide a corresponding p-value, the analysis is useful for providing an exploratory view of the potential underlying changes in the spatial distribution of case reporting.

Anthrax is a neglected zoonosis that is often prone to underreporting in livestock, wildlife and humans [8], [12], [52]. The observed decline over time in this study may be related to improved control measures, or could be a result of underreporting. The data collected here were obtained through passive surveillance, which can be subject to systematic error, relying heavily on the accuracy and timely dissemination of data from health facilities and may be subject to healthcare facility admission bias. Districts in Azerbaijan that provide better access to healthcare facilities could potentially have higher rates of reporting. In Azerbaijan, there is an underutilization of the nation's healthcare in rural areas that could skew reporting towards more urbanized districts [53]. Aggregating the data may have influenced the spatial distribution of risk and clustering observed in this study. However, our analysis of yearly incidence using the mean center of reporting revealed a similar pattern, thereby corroborating the spatial shift in reporting and clustering identified by SaTScan. The eastward shift in reporting may have also been influenced by the annexation of land in western Azerbaijan by the Nargorno-Karabakh Republic. Current disease reports are not routinely collected in this region and may bias reporting to the east. Inference on changes in control measures is limited due to the fact that data on livestock vaccination were only available for the period of preemptive vaccination, and although reactive vaccination was regularly carried out during Soviet governance in response to outbreaks it is not clear to what extent this took place. The breakpoint in our segmented regression model indicated a declining incidence around the initiation of vaccination, however, higher resolution data and future research incorporating models such as Zinsstag et al. [20], [54] are needed to link improvements in human incidence to animal vaccination while also estimating the economic benefits to public health.

While the true cause of the changing patterns of incidence is unknown, it is likely due to a combination of factors related to the Soviet collapse, subsequent improvements in socio-economic indicators, changes in agricultural production, and recent investments in public health infrastructure. *B. anthracis* is able to survive in the environment for long periods of time, possibly years, and periods of quiescence require sustained routine vaccination in endemic areas [8]. A recent outbreak of more than 600 human anthrax cases in Bangladesh after a 20 year absence has reiterated the importance of continued vigilance [43]. There is a critical need for researchers to construct dynamic transmission models that attempt to link the benefits of interventions such anthrax vaccination to public health. Our findings can serve as a basis for highlighting the potential impacts that additional “One Health” studies can have on better addressing neglected disease such as anthrax. Future research should focus on collecting individual level data on human and livestock anthrax following Roth et al. [19] and Zinsstag et al. [54] to better formulate public health interventions for anthrax in Azerbaijan and the neighboring countries. The changing patterns of anthrax identified here illustrates the need for dynamic surveillance that takes into account alterations in the distribution of disease over time [55]. Areas of high incidence identified in this study can be used to target interventions such as livestock vaccination, veterinary outreach, and education campaigns targeting the human population at risk. Our findings highlight the potential impacts governmental change and policy can have on the control of zoonotic diseases such as anthrax and underlines the need for addressing the impacts in other former Soviet states.

## Supporting Information

Figure S1 Boxplots show the differences in the latitudinal and longitudinal position of the mean center between time periods. The mean center is describing the concentration of reporting by district during the Soviet (1984–1991), post-Soviet (1992–1995), and), and preemptive livestock vaccination (1996–2010) periods. Graph shows a higher concentration in the west during the Soviet period and more northern concentration during preemptive livestock vaccination.
(TIF)
