# Supplementary figures and images for: Changing Patterns of Human Anthrax in Azerbaijan during the Post-Soviet and Preemptive Livestock Vaccination Eras
Source: PLoS Negl Trop Dis. 2014 Jul 17;8(7):e2985. doi: 10.1371/journal.pntd.0002985 (PMC4102439; doi:10.1371/journal.pntd.0002985)

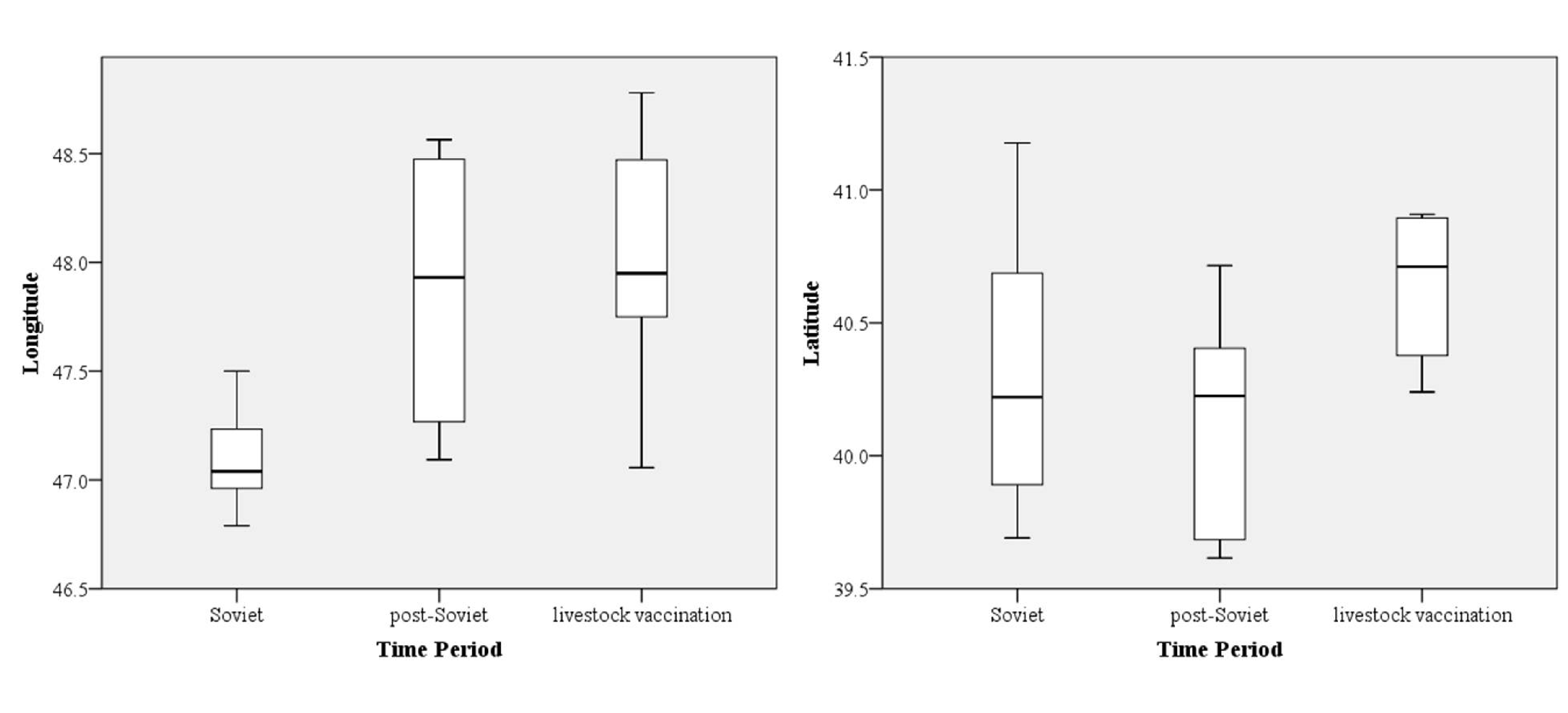

Supplement: Figure S1 — Boxplots show the differences in the latitudinal and longitudinal position of the mean center between time periods. The mean center is describing the concentration of reporting by district during the Soviet (1984–1991), post-Soviet (1992–1995), and), and preemptive livestock vaccination (1996–2010) periods. Graph shows a higher concentration in the west during the Soviet period and more northern concentration during preemptive livestock vaccination. (TIF) [file pntd.0002985.s001.tif]
